# Development of an HbA1c-Based Conversion Equation for Estimating Glycated Albumin in a Korean Population with a Wide Range of Glucose Intolerance

**DOI:** 10.1371/journal.pone.0095729

**Published:** 2014-04-22

**Authors:** Chang Hee Jung, You-Cheol Hwang, Kwang Joon Kim, Bong Soo Cha, Cheol-Young Park, Won Seon Jeon, Jae Hyeon Kim, Sang-Man Jin, Sang Youl Rhee, Jeong-taek Woo, Byung-Wan Lee

**Affiliations:** 1 Department of Internal Medicine, Asan Medical Center, University of Ulsan College of Medicine, Seoul, Korea; 2 Department of Internal Medicine, Kyung Hee University School of Medicine, Seoul, Korea; 3 Department of Internal Medicine, Severance Hospital, University of Yonsei University College of Medicine, Seoul, Korea; 4 Department of Internal Medicine, Kangbuk Samsung Hospital, Sungkyunkwan University School of Medicine, Seoul, Korea; 5 Department of Internal Medicine, Samsung Medical Center, Sungkyunkwan University School of Medicine, Seoul, Korea; University of North Carolina at Chapel Hill, United States of America

## Abstract

**Background:**

Compared to the golden standard glycation index of HbA1c, glycated albumin (GA) has potentials for assessing insulin secretory dysfunction and glycemic fluctuation as well as predicting diabetic vascular complications. However, the reference ranges of GA and a conversion equation need to be clearly defined. We designed this study to determine the reference ranges in patients with normal glucose tolerance (NGT) based on conventional measures of glycemic status and to devise a conversion equation for calculating HbA1c and GA in a Korean population.

**Methodology/Principal Findings:**

In this multicenter, retrospective, cross-sectional study, we recruited antidiabetic drug-naïve patients with available glycemic variables including HbA1c, GA, and fasting plasma glucose regardless of glucose status. For the reference interval of serum GA, 5^th^ to 95^th^ percentile value of GA in subjects with NGT was adopted. The conversion equation between HbA1c and GA was devised using an estimating regression model with unknown break-points method. The reference range for GA was 9.0–14.0% in 2043 subjects. The 95^th^ percentile responding values for FPG, and HbA1c were approximately 5.49 mmol/l, and 5.6%, respectively. The significant glycemic turning points were 5.868% HbA1c and 12.2% GA. The proposed conversion equation for below and above the turning point were GA (%) = 6.960+0.8963 × HbA1c (%) and GA (%) = −9.609+3.720 × HbA1c (%), respectively.

**Conclusions/Significance:**

These results should be helpful in future studies on the clinical implications of high GA relative to HbA1c and the clinical implementation of diabetes management.

## Introduction

Currently, HbA1c is the golden standard glycation index for use in clinical practice and research [1]. However, it can be unreliable in conditions affecting the lifespan of erythrocytes as well as the clinical state in which glycemic control alleviates or deteriorates in the short period. By overcoming the shortcomings of HbA1c, glycated albumin (GA) has gained popularity as an useful index for intermediate glycation and pathogenic protein [2].

Besides the role of GA as an intermediate glycation index, several previous studies have suggested additional values of GA in reference to HbA1c levels in assessing insulin secretory dysfunction and fluctuating glycemic excursions [2]–[4]. Furthermore, elevated serum GA levels as well as GA/HbA1c ratio have been suggested to predict diabetic macrovascular complications [5]. Therefore, a simple and accurate conversion equation determining GA using HbA1c (and vice versa) would help physician for managing patients with diabetes, although it remains to be clarified. Previously, an easy but rough approximation (i.e., HbA1c = GA/3) has been suggested [6]. However, GA levels are at unexpectedly high levels, over HbA1c in patients with long duration of diabetes or decreased insulin secretory function. Hence, this equation was not acceptable for empirical adoption and lacked statistical significance [7]. In addition, a similar but different concept of the GA/HbA1c ratio confers additional clinical implications regarding glucometabolic homeostasis and diabetic atherosclerosis rather than simply converting GA to HbA1c [2], [3], [5].

Based on a previous study, where GA reportedly increased by 2.5–3.2% for every 1% increase in serum HbA1c (range: 6.5–14.0% HbA1c) [4], we hypothesized that the proportion of HbA1c to GA would differ in prediabetic and diabetic patients depending on their glycemic status such as normal glucose tolerance (NGT), pre-diabetes and type 2 diabetes (T2D). In this multicenter cross-sectional study, our aims are to determine the reference ranges in patients with NGT based on conventional measures of glycemic status [8], [9], and devise a conversion equation for calculating HbA1c and GA in a Korean population.

## Materials and Methods

### Ethics Statement

This study was approved by the independent ethics committee/institutional review board (IRB) at each study site (IRB of Severance Hospital Yonsei University College of Medicine, Asan Medical Center, University of Ulsan College of Medicine, Kyunghee University Hospital, Kyunghee University Hospital at Gangdong, Kangbuk Samsung Medical Center, Sungkyunkwan University School of Medicine, and Samsung Medical Center, Sungkyunkwan University School of Medicine, respectively). All enrolled subjects provided written informed consent.

### Study Population

Study subjects were recruited from outpatient clinics at 6 major referral centers; a total of 2450 patients who were registered with the health check-up program of Severance Hospital or the Newly Detected Diabetes Registry in Asan Medical Center, Kyunghee Hospital, Kangdong Kyunghee Hospital, Kangbuk Samsung Hospital, Samsung Medical Center, and Severance Hospital were recruited. All patients had their GA, HbA1c, and fasting plasma glucose (FPG) levels measured. All study participantshad fasting C-peptide levels of >0.5 ng/mL. We excluded patients without GA, HbA1c, or FPG data and those with any of the following criteria that might affect GA or HbA1c: hemoglobin (Hb) level <12 g/dL for women and <13 g/dL for men; chronic kidney disease ≥ stage 3 (i.e., estimated glomerular filtration rate [eGFR] <60 mL/minute/1.73 m^2^ according to the Modification of Diet in Renal Disease criteria [MDRD]); active thyroid disease; liver cirrhosis; or nephrotic syndrome.

### Clinical and Laboratory Examination

The body mass index (BMI) was calculated as weight in kilograms divided by the square of height in meters. All blood samples were obtained in the morning, following an overnight fasting of at least 12 hours. Plasma glucose was measured by the hexokinase method. Lipid parameters, including serum total cholesterol, triglycerides, HDL cholesterol (HDL-C), directly-measured LDL cholesterol (LDL-C) levels, and liver enzymes, including aspartate aminotransferase (AST), alanine aminotransferase (ALT), and gamma-glutamyltransferase (GGT), were measured using an enzymatic colorimetric method. Serum GA was determined by an enzymatic method using an albumin-specific proteinase, ketoamine oxidase and albumin assay reagent (LUCICA GA-L, Asahi Kasei Pharma Co., Tokyo, Japan), and a Hitachi 7699 Pmodule autoanalyzer (Hitachi Instruments Service, Tokyo, Japan). The coefficient of variation (CV) was 1.43%. HbA1c was measured by high-performance liquid chromatography (HPLC) method. The reference intervals of HbA1c were between 4.0% and 6.0%. HOMA of insulin resistance (HOMA-IR) was calculated as the product of fasting serum insulin (µU/ml) and FPG (mmol/l) concentrations, divided by 22.5. HOMA of beta cell function (HOMA-beta) was calculated according to the equation: HOMA-beta (%) = (20×fasting serum insulin)/(FPG-3.5). All enzyme activities were measured at 37°C.

In this study, NGT was defined by the previously defined criteria [8], [9]: (1) FPG <5.55 mmol/l and (2) HbA1c <5.7%.

### Statistical Methods

Continuous variables are expressed as the mean (± standard deviation [SD]). Categorical variables are expressed as proportions (%). Demographic and biochemical characteristics of the study population between the two arbitrarily defined groups in this study were compared using independent t-test for continuous variables and the chi-squared test for categorical variables. To determine the relationship between serum GA and HbA1c levels according to the deterioration of glucose tolerance, we performed the estimating regression model with unknown break-points as previously described [10]. The normal reference interval of serum GA was determined directly from the percentage of interest (i.e., the 5–95^th^ percentile of patients with NGT). The relationship between glycemic parameters including FPG, GA, and HbA1c were assessed by Pearson correlation analysis. The relevant values of HbA1c and GA corresponding to specific FPG concentrations were calculated using linear regression analysis after assigning HbA1c and GA as dependent variables and FPG as independent variables. R version 2.14.0 (R Foundation for Statistical Computing, Vienna, Austria, http://www.R-project.org) was used to analyze the data. All *p*-values <0.05 were considered statistically significant.

## Results

**Figure 1:**
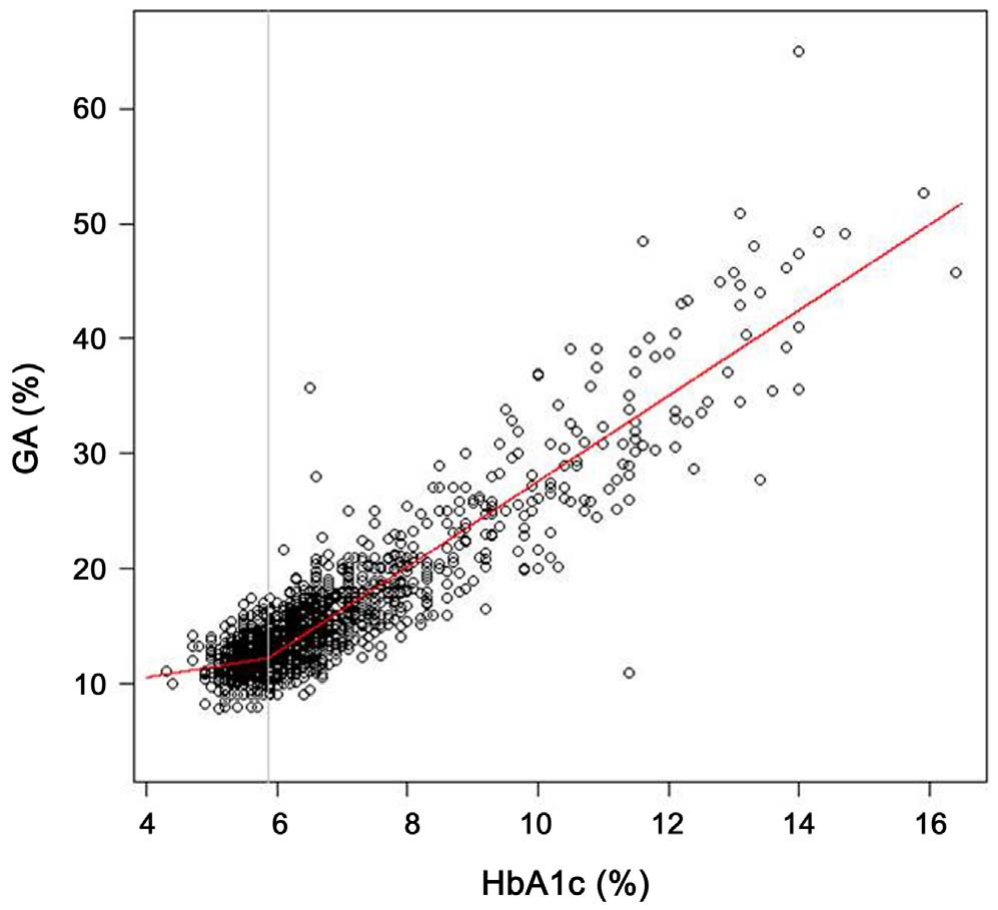
Estimating regression model analysis with unknown break-points.

A total of 2043 subjects out of all that were recruited satisfied the inclusion and exclusion criteria of this study. The clinical characteristics of the study participants are shown in Table 1. The mean age of the study subjects was 54.1±11.1 years with relatively even gender distribution (57.5% male). The mean BMI was 25.0±3.3 kg/m^2^. When we fitted estimating regression models with unknown break-points, serum GA levels were drastically increased from HbA1C level of 5.868% (Fig. 1). Based on this turning point of the slope, we classified the subjects into two groups [group I, HbA1c <5.9% (n = 736); group II, HbA1c ≥5.9% (n = 1307)]. The subjects in group II were statistically older (50.9±10.4 *vs.* 56.0±11.0 years years in group I and group II, respectively; *p*<0.001) and more obese (24.2±3.2 *vs.* 25.4±3.2 kg/m^2^ years in group I and group II, respectively; *p*<0.001). The Hb concentration were similar between the two groups (14.6±1.2 *vs.* 14.6±1.3 g/dL years in group I and group II, respectively; *p* = 0.949). Similar to our previous reports [3], [4], serum albumin levels were different between the two groups (4.5±0.4 *vs.* 4.4±0.3 mg/dL in group I and group II, respectively; *p*<0.001). The percentage of current smoker was similar (22.8 *vs.* 21.0% dL in group I and group II, respectively; *p* = 0.391), but the blood pressure and glucometabolic profiles, including FPG, GA, HOMA-IR, and HOMA-β, were significantly higher in group II. Finally, significantly more patients in group II were taking antihypertensive and antilipid medications.

**Table 1:** Clinical and biochemical characteristics of the study participants.

	Total	Group I (GI)	Group II (GII)	*P* value
Variables	(n = 2043)	(n = 736)	(n = 1307)	(GI vs. GII)
Male (n, %)	1175 (57.5)	395 (53.7)	780 (59.7)	0.009
Age (years)	54.1 (11.1)	50.9 (10.4)	56.0 (11.0)	<0.001
BMI (kg/m^2^)	25.0 (3.3)	24.2 (3.2)	25.4 (3.2)	<0.001
WC (cm)	85.5 (8.9)	83.2 (8.7)	87.1 (8.6)	<0.001
Systolic BP (mmHg)	126.5 (16.4)	121.6 (15.1)	129.2 (16.4)	<0.001
Diastolic BP (mmHg)	78.0 (11.0)	76.2 (10.4)	78.9 (11.1)	<0.001
Current smoker (%)	17.0	22.8	21.0	0.391
Anti-HTN medication (%)	23.7	18.7	32.0	<0.001
Anti-lipid medication (%)	14.2	15.0	21.1	0.002
FPG (mmol/l)	6.67 (2.09)	5.58 (0.67)	7.29 (2.36)	<0.001
HbA1c (%)	6.6 (1.6)	5.5 (0.2)	7.3 (1.7)	<0.001
GA (%)	15.4 (6.3)	11.9 (1.5)	17.4 (7.0)	<0.001
Hb (g/dl)	14.6 (1.3)	14.6 (1.2)	14.6 (1.3)	0.949
Protein (g/l)	7.1 (0.4)	7.0 (0.4)	7.1 (0.4)	0.001
Albumin (g/l)	4.5 (0.4)	4.5 (0.4)	4.4 (0.3)	<0.001
AST (U/l)	25 (15)	23 (10)	27 (18)	<0.001
ALT (U/l)	28 (27)	24 (15)	31 (31)	<0.001
GGT (U/l)	41 (46)	38 (48)	44 (44)	0.062
Total cholesterol (mmol/l)	4.95 (1.04)	4.94 (0.92)	4.96 (1.10)	0.636
Triglycerides (mmol/l)	1.66 (1.36)	1.42 (0.96)	1.79 (1.52)	<0.001
HDL-C (mmol/l)	1.35 (0.47)	1.39 (0.35)	1.34 (0.51)	0.009
LDL-C (mmol/l)	2.93 (0.93)	3.00 (0.82)	2.89 (0.99)	0.005
eGFR (ml/min/1.73 m^2^)	93.6 (23.6)	96.2 (28.1)	92.3 (21.0)	0.006
HOMA-IR	2.7 (2.3)	1.9 (1.2)	3.2 (2.6)	<0.001
HOMA-β (%)	69.1 (52.4)	76.1 (49.4)	65.0 (53.8)	<0.001

Table 2 shows the selected percentile concentrations for FPG, GA, and HbA1c in patients with NGT. The 25–75^th^ percentile interval value was approximately 11.0–12.5% for GA, while the 5–95^th^ percentile interval was approximately 9.0–14.0% in patients with NGT. In this study, the 95^th^ percentile values for FPG, GA, and HbA1c were approximately 99 mg/dL, 14.0%, and 5.6%, respectively. The FPG and HbA1c values, according to the individual percentile points, were almost exactly same between sexes; but, the GA values were similar.

**Table 2:** Means and selected percentiles of GA, FPG, HbA1c, HOMA-IR and HOMA-β levels in subjects with NGT according to the sex.

Variables	Mean (SD)	Percentile				
		5^th^	25^th^	50^th^	75^th^	95^th^
GA (%)						
* Total*	11.5 (1.4)	9.0	11.0	11.3	12.5	14.0
* Men*	11.5 (1.2)	10.0	11.0	11.1	12.1	13.5
* Women*	11.6 (1.6)	9.0	10.9	11.4	12.5	14.2
FPG (mmol/l)						
* Total*	5.03 (0.37)	4.27	4.83	5.11	5.33	5.49
* Men*	5.04 (0.37)	4.24	4.83	5.11	5.33	5.49
* Women*	5.03 (0.37)	4.25	4.83	5.11	5.33	5.49
HbA1c (%)						
* Total*	5.4 (0.2)	5.0	5.4	5.5	5.6	5.6
* Men*	5.4 (0.2)	5.0	5.4	5.5	5.6	5.6
* Women*	5.4 (0.2)	5.0	5.3	5.5	5.6	5.6
HOMA-IR						
* Total*	1.4 (0.9)	0.5	0.8	1.2	1.8	3.0
* Men*	1.5 (1.1)	0.4	0.8	1.2	1.9	4.3
* Women*	1.4 (0.7)	0.6	0.8	1.2	1.8	2.6
HOMA-β (%)						
* Total*	85.3 (62.5)	28.5	47.2	70.9	100.1	213.9
* Men*	85.3 (59.6)	21.1	48.6	70.7	99.5	224.9
* Women*	85.4 (64.8)	31.4	45.7	71.6	101.5	190.4

The number of men, and women tested were 107, and 155, respectively.

**Figure 2:**
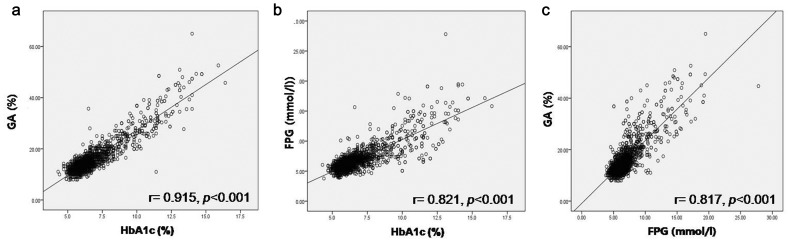
Correlation between HbA1c and GA (A), FPG and HbA1c (B), and FPG and GA (C).

Figure 2 shows the overall correlations between HbA1c and GA (Fig. 2A), FPG and HbA1c (Fig. 2B), and FPG and GA (Fig. 2C). The positive correlation coefficient between HbA1c and GA was the highest (*r* = 0.915; *p*<0.001; Fig. 2A). Furthermore, FPG concentrations demonstrated significant positive correlations to both HbA1c (*r* = 0.821; *p*<0.001, Fig. 2B) and GA (*r* = 0.817; *p*<0.001, Fig. 2C). Similar to previous studies [3], [4], our results indicated that FPG demonstrated a stronger correlation with HbA1c over GA. The HbA1c and GA levels corresponding to FPG were 5.9% and 12.6% for 5.55 mmol/l FPG, 6.3% and 14.0% for 6.11 mmol/l, and 6.8% and 16.0% for 6.94 mmol/l, respectively.

Figure 1 shows the significant glycemic turning point, which demonstrates an apparently linear trend above and below the turning point from normal glucose to high glucose status. As previously mentioned, we divided the patients into two groups based on this turning point. Below and above 5.868% HbA1c, the conversion equations obtained using the estimating regression models with unknown breakpoints [10] for group I (HbA1c<5.868%) and group II (HbA1c≥5.868%) were GA (%) = 6.960+0.8963 ×HbA1c (%) and GA (%) = −9.609+3.720×HbA1c (%), respectively. The positive correlation coefficients for HbA1c and GA were 0.135 (*p*<0.001) and 0.912 (*p*<0.001) for groups I and II, respectively.

In table 3, the mean values of GA, GA/HbA1c ratio, and FPG were analyzed according to the levels of HbA1c, which is the standard glycation index used in clinical practice and research. Based on the result regarding the turning point of 5.9% HbA1c in the continuous plots of GA and HbA1c (Fig. 1), and 6.5% HbA1c, a well-known cut-off value for the diagnosis of diabetes [8], [9], we adopted both 5.9% and 6.5% HbA1c as reference points for use in this study. In the ranges of non-diabetes, mean values of GA/HbA1c ratio (2.16 to 2.17) were similar. In the ranges of diabetes, however, the mean values of GA/HbA1c ratio ranged from 2.34 to 3.17. Similar to previous study [4], which included patients with T2D who were receiving medications, the mean GA/HbA1c ratio (2.48–3.13) increased as HbA1c increased.

**Table 3:** Mean values of glycemic parameters according to HbA1c.

HbA1c (%)	N (%)	HbA1c (%)	GA (%)	GA/A1c	FPG (mmol/l)
HbA1c<5.9	736 (36.0)	5.5	11.9	2.16	5.58
5.9≤HbA1c<6.5	575 (28.1)	6.1	13.3	2.17	6.04
6.5≤HbA1c<7.5	370 (18.1)	6.8	16.0	2.34	6.90
7.5≤HbA1c<8.5	143 (7.0)	7.8	18.7	2.38	7.80
8.5≤HbA1c<9.5	71 (3.5)	8.9	23.6	2.65	9.14
9.5≤HbA1c<10.5	49 (2.4)	10.0	26.9	2.70	9.97
10.5≤HbA1c<11.5	41 (2.0)	11.0	29.7	2.71	11.47
11.5≤HbA1c<12.5	29 (1.4)	11.8	35.6	3.02	11.53
HbA1c≥12.5	29 (1.4)	13.6	43.3	3.17	15.25

## Discussion

Current clinical guidelines for assessment of glycemic control recommend self-monitoring of blood glucose (SMBG) by patient and HbA1c as a part of hospital continuing care [8], [9]. The latter is thought to reflect the average glycemia over a few months and has a strong predictive value for diabetes complications demonstrated in the large-scale studies such as the Diabetes Control and Complications Trial (DCCT) and U.K. Prospective Diabetes Study (UKPDS) [11]–[13]. However, notwithstanding the effects of erythrocyte turnover (hemolysis, blood loss) and hemoglobin variants [6], HbA1c is limited because it does not provide a measure of glycemic variability or hypoglycemia especially in patients with both T2D and severe insulin deficiency [8]. Because of these challenges, it is recommended for glycemic control to be judged by the combination of the results of SMBG and the HbA1c for patients prone to glycemic variability [8], [14]. These unmet needs have allowed GA to gain popularity among physicians. Growing evidence demonstrates that GA, in conjunction with the GA/HbA1c ratio might be more accurate than HbA1c alone for assessing insulin secretory dysfunction, which resulted in glycemic fluctuation and variability [2]–[4] and can be used to predict atherosclerosis [5], [15]. Considering these potentials of GA, accurate interpretation of GA on the basis of HbA1c such as increased GA/HbA1c ratio or unexpected high GA levels over the HbA1c one is of paramount importance for assessing the pathophysiologic changes [2]–[4] and predicting the diabetic complications in patients with T2D [5]. However, an accurate conversion equation that takes into account the GA and HbA1c values (and vice versa) has not been developed for use in Korean populations.

The aim of the present study was to develop a simple equation for converting HbA1c to GA in a Korean population. By establishing expected GA levels from HbA1c, we could get additional information on the glycemic fluctuation, insulin secretory dysfunction and pro-atherogenic condition in subjects with T2D in whom the laboratory levels of GA would be above the calculated GA levels. The present study demonstrates 3 main findings. First, the 5.0^th^–95.0^th^ percentile reference interval for GA was 9.0% to 14.0% while adopting the cut-off values of impaired glucose tolerance of both FPG levels ≥5.55 mmol/l, and HbA1c levels ≥5.7%. Second, we noted a significant glycemic turning point at 5.868% HbA1c and an apparently linear slope (both below and above this point) on continuous plots for GA and HbA1c in patients with various levels of glucose intolerance (the corresponding GA value was 12.2%). Third, we devised the following conversion equations for GA and HbA1c in groups I (HbA1c<5.868%) and II (HbA1c≥5.868%):GA (%) = 6.960+0.8963 ×HbA1c (%) and GA (%) = −9.609+3.720×HbA1c (%), respectively.

Regarding the reference values for GA, it ranged between 11.9–15.8% (mean ±2 SD) in a healthy American population of both white and black patients without a known history of diabetes in North Carolina (n = 201 patients); this range was determined using the 75-g oral glucose tolerance test (OGTT) and demonstrated a significant racial difference [16]. In a study with a limited number of Italian patients (n = 32), the GA of the normal control group ranged between 11.7–16.9% (2.5–97.5^th^ percentile) [17]. In a study conducted by the Japan Diabetes Society, the reference range for GA determined using 75-g OGTT in a selected Japanese reference population (n = 699 patients) was 12.3–16.9% (mean ±2 SD) [18]. In Chinese patients with NGT (n = 380), the 2.5–97.5^th^ percentile was 10.8–17.1% GA [19]. On the other hand, the reference range (9.0–14.0% GA corresponding to the 5–95^th^percentile) in this population of Korean patients (n = 2043) did not exactly correspond to other Asian ethnicities, including Japanese and Chinese populations. This could be explained by the different FPG cutoff values that were used to define NGT (<110 mg/dL in Japanese population) [18] and the fact that 75-g OGTT was not used to assess our Korean population. In addition, we included patients with both FPG <5.55 mmol/l and HbA1c <5.7%. Similar to the racial differences noted in the American population, ethnic differences are debatable and require further investigations. Furthermore, there are no international standards for GA or any external quality assessment programs. Therefore, international standardization is clearly required for use in both clinical practice and research.

We used 2 steps to develop our conversion equation. In the first step, we identified a significant glycemic turning point (5.868% HbA1c; Fig. 1) using the continuous plots of GA and HbA1c. In the second step, we calculated separate conversion equations for both above and below this turning point. A plausible explanation for this turning point might be the inert structural characteristics and different glycation processes of both albumin and Hb. Serum albumin is directly exposed to high glucose levels, while Hb, which resides within erythrocytes, is indirectly exposed to high glucose. Previous studies reported that the *in vivo* nonenzymatic glycation rate of albumin is approximately 9 times that of human Hb [20], and albumin glycation proceeds 10 times more quickly than Hb glycation [21]; these findings could partially account for the turning point [22]. In addition, glycemic fluctuations, which are observed in patients with high glucose status and decreased insulin secretory function [3], [4] affect plasma proteins (such as albumin) more easily than intracellular proteins (such as Hb); this can result in higher GA levels, even in newly diagnosed T2D patients. As far as we know, this might be the first attempt to devise a conversion equation for GA and HbA1c based on the glycemic turning point. The proposed conversion equation (GA = −9.609+3.720 × HbA1c [%]) for newly diagnosed T2D patients corresponds well to our previously reported equation (GA = −8.01+3.66 ×HbA1c [%]) [4].

There are a few important limitations to our study that warrant consideration, besides its retrospective nature. First, the lack of 2-hour OGTT is a limitation because it may have resulted in the inclusion of patients with impaired glucose tolerance to the NGT group. Second, although we measured plasma GA at each hospital under the regulation of the US National Glycohemoglobin Standardization Program, it would be better if the GA measurements were performed by single central laboratory. Lastly, this study included only Korean patients, preventing generalization with other ethnic populations.

The present study suggests a reference range of 9.0–14.0% GA for Korean patients. Based on the continuous plots of GA and HbA1c in patients with normal and high glucose status, the significant glycemic turning points are 5.868% HbA1c and 12.2% GA. The proposed conversion equations below and above the turning point are GA (%) = 6.960+0.8963 × HbA1c (%) and GA (%) = −9.609+3.720 × HbA1c (%), respectively. Using the equation that we proposed, the differences between measured GA level and calculated GA level could be identified. These results should be helpful in future studies on investigating the clinical implication of GA as a glycemic index in specific patients with unexpected high GA levels over the HbA1c and the clinical implementation of diabetes management using GA.
